# Handgrip strength and all‐cause dementia incidence and mortality: findings from the UK Biobank prospective cohort study

**DOI:** 10.1002/jcsm.12857

**Published:** 2022-04-21

**Authors:** Irene Esteban‐Cornejo, Frederick K. Ho, Fanny Petermann‐Rocha, Donald M. Lyall, David Martinez‐Gomez, Verónica Cabanas‐Sánchez, Francisco B. Ortega, Charles H. Hillman, Jason M.R. Gill, Terence J. Quinn, Naveed Sattar, Jill P. Pell, Stuart R. Gray, Carlos Celis‐Morales

**Affiliations:** ^1^ PROFITH “PROmoting FITness and Health through physical activity” Research Group, Sport and Health University Research Institute (iMUDS), Department of Physical Education and Sports, Faculty of Sport Sciences University of Granada Granada Spain; ^2^ BHF Glasgow Cardiovascular Research Centre, Institute of Cardiovascular and Medical Sciences University of Glasgow Glasgow UK; ^3^ Institute of Health and Wellbeing University of Glasgow Glasgow UK; ^4^ Faculty of Medicine Universidad Diego Portales Santiago Chile; ^5^ Department of Preventive Medicine and Public Health Autonomous University of Madrid/IdiPaz, CIBER of Epidemiology and Public Health (CIBERESP) Madrid Spain; ^6^ IMDEA Food Institute, CEI UAM + CSIC Madrid Spain; ^7^ Faculty of Sport and Health Sciences University of Jyväskylä Jyväskylä Finland; ^8^ Department of Psychology Northeastern University Boston MA USA; ^9^ Department of Physical Therapy, Movement & Rehabilitation Sciences Northeastern University Boston MA USA; ^10^ Centre for Research in Exercise Physiology (CIFE), Universidad Mayor Santiago Chile; ^11^ Human Performance Laboratory, Research Group in Education, Physical Activity and Health (GEEAFyS) Catholic University of Maule Talca Chile

**Keywords:** Alzheimer, Vascular dementia, Muscular strength, Prevention, Adults, Mortality

## Abstract

**Background:**

This study aimed to investigate the associations of grip strength with incidence and mortality from dementia and whether these associations differ by sociodemographic and lifestyle factors.

**Methods:**

A total of 466 788 participants of the UK Biobank (median age 56.5 years, 54.5% women). The outcome was all‐cause dementia incidence and mortality and the exposure was grip strength. Grip strength was assessed using a Jamar J00105 hydraulic hand dynamometer.

**Results:**

Excluding the first 2 years of follow‐up (landmark analysis), mean follow‐up was 9.1 years (inter‐quartile range: 8.3; 9.7) for incidence and 9.3 (inter‐quartile range: 8.7; 10.0) for mortality. During this time, 4087 participants developed dementia, and 1309 died from it. Lower grip strength was associated with a higher risk of dementia incidence and mortality independent of major confounding factors (*P* < 0.001). Individuals in the lowest quintile of grip strength had 72% [95% confidence interval (CI): 1.55; 1.92] higher incident dementia risk and 87% [95% CI: 1.55; 2.26] higher risk of dementia mortality compared with those in the highest quintile. Our PAF analyses indicate that 30.1% of dementia cases and 32.3% of dementia deaths are attributable to having low grip strength. The association between grip strength and dementia outcomes did not differ by lifestyle or sociodemographic factors.

**Conclusions:**

Lower grip strength was associated with a higher risk of all‐cause dementia incidence and mortality, independently of important confounding factors.

## Introduction

Dementia is a growing public health concern worldwide and has huge social and economic impacts. The total annual cost of dementia worldwide is estimated to be $US1 trillion and is projected to rise to $US2 trillion by 2030. There are currently around 50 million people with dementia in the world, with over 9.9 million new cases each year, having severely detrimental effects on the affected individuals and their families.^1^, ^2^ It is, therefore, critical to improve our understanding of risk factors, which can predict dementia, for which prospective, population‐based studies can be of help. Although, several modifiable risk factors, including body mass index, smoking, poor diet, and physical activity, have been shown to be associated with dementia,^3^ there is limited evidence on markers of physical capability such as muscular strength.^4^, ^5^, ^6^

Handgrip strength is a cheap and easy to measure marker of general health in middle age and older adulthood.^7^, ^8^, ^9^, ^10^ Low grip strength has been associated with higher risk of developing cardiovascular diseases (CVDs), respiratory diseases, cancer, and all‐cause mortality across several cohorts, age groups, and countries.^11^, ^12^, ^13^, ^14^ Recent studies have also indicated that handgrip strength is an emerging marker of brain health and cognitive decline representing a correlate of measure of neural function.^15^, ^16^ For example, lower handgrip strength has been associated with cognitive decline, hippocampal atrophy, and white matter lesions in both general and clinical populations.^15^, ^17^, ^18^, ^19^

Interestingly, while muscle strengthening exercises might slow cognitive decline,^20^ very few studies have examined the association of handgrip strength with the risk of dementia,^4^, ^5^, ^6^ and so far no studies have investigated associations with mortality from all‐cause dementia. In particular, two studies showed handgrip strength as a predictor of incident Alzheimer disease,^4^, ^5^ while another study found that lower handgrip strength was associated with an increased risk of dementia.^6^ Collectively, these studies have been conducted in very specific populations that largely differ from the general population (e.g. Catholic clergy members), relatively small sample sizes (i.e. *n* = 877 to 2288), or were restricted to participants aged 65 years and older. Dementia and other neurodegenerative diseases are preceded by a ‘silent’ clinical period that can be longer than a decade.^1^ This highlights the importance of examining such associations both in mid‐life and late‐life and determining how associations vary with age and other relevant health‐related and lifestyle behavioural moderators. By using the UK Biobank study, one of the largest prospective cohort studies in the UK, we had the opportunity to overcome many of the limitations of previous studies regarding sample size and population characteristics as well as assess whether the association between handgrip strength and dementia varied by sociodemographic, health‐related, and lifestyle behavioural factors.

Accordingly, the aim of this study was to examine the association between handgrip strength and all‐cause dementia incidence and mortality,^21^ and how these associations varied with sociodemographic, health‐related, and lifestyle behavioural factors in the UK Biobank cohort.

## Methods

### Study design

UK Biobank is a population‐based cohort of approximately 500 000 participants (5.5% response rate) aged 37–73 years from the UK.^22^ Baseline assessments took place between April 2007 and December 2010 as described in detail elsewhere.^23^, ^24^

The main outcomes for this study were incidence and mortality from all‐cause dementia, Alzheimer, and vascular dementia. The exposure variable was handgrip strength. The covariates were sociodemographic factors (age, sex, ethnicity, and deprivation index), health‐related factors (body mass index categories, multimorbidity and long‐standing illness), and lifestyle behaviours (walking pace, sleep duration, watching TV, smoking and dietary intake including alcohol, fruits and vegetables, red meat, processed meat, and oily fish intake). The present study included 466 830 participants who had available data on dementia outcomes.

### Procedures

#### All‐cause dementia incidence and mortality

Death certificates held by the National Health Service (NHS) Information Centre (England and Wales) and the NHS Central Register Scotland (Scotland) were used to obtain date of death. Record linkage to Health Episode Statistics (England and Wales) and to the Scottish Morbidity Records 01 (Scotland) was used to identify date and cause of hospital admissions. Detailed information regarding the linkage procedure can be found online (http://www.ic.nhs.uk/services/medical‐research‐informationservice).

Mortality data for the full cohort were available up to 1 June 2020 at the time of analysis and so the current analysis of all‐cause dementia mortality was censored at this date or date of death if this occurred earlier. Incident dementia included fatal and non‐fatal dementia cases, which were extracted from hospital admission records available until 1 June 2020 for the full UK Biobank cohort. Follow‐up was censored at the date of incident dementia or all‐cause death if this occurred earlier. All‐cause dementia was defined as an ICD‐10 (International Classification of Diseases, 10th revision) codes F00 (dementia in Alzheimer disease), F01 (vascular dementia), F02 (dementia in other diseases) and F03 (unspecified dementia).^21^ Our analyses excluded the 124 participants with self‐reported diagnosed all‐cause dementia, Alzheimer's or cognitive impairment at baseline.

#### Handgrip strength

Handgrip strength was assessed using a Jamar J00105 hydraulic hand dynamometer. This measures grip force isometrically (without movement), and can be adjusted for hand size in five half‐inch increments. The dual‐scale readout displays isometric grip force from 0 to 90 kg, with a ‘peak‐hold’ needle that remains in place once grip is released. The dynamometer was calibrated before each measurement day. The participant was sat upright in a chair and place their forearms on armrests. The participant's elbow of the arm holding the dynamometer was against their side and bent to a 90° angle so that their forearm is pointing forwards with their thumb uppermost. Their wrist was straight so that their hand was either pointing forwards or bent slightly outwards. The participant was asked to squeeze the handle of the dynamometer as strongly as they can for about 3 s, and encouragement was given while doing so. Right‐hand and left‐hand measurements were recorded. A previous analysis of UK Biobank data found that the ability of handgrip strength to predict mortality and health‐related outcomes was similar in absolute or relative terms and recommended the use of absolute units (kilogrammes) for clinical utility.^25^ Therefore, the mean of the right and left values was expressed in absolute units (kilogrammes) for subsequent analysis. Handgrip strength was also treated as sex‐age‐specific quintiles of handgrip strength as well as 5 kg lower handgrip strength.

#### Sociodemographic factors

Age was calculated from dates of birth and baseline assessment. Sex and ethnicity were self‐reported at baseline. Area‐based socioeconomic status was derived using the Townsend deprivation index.^26^

#### Health‐related factors

Body height and weight were measured by trained nurses during the baseline assessment. Body mass index (BMI) was calculated as weight (kilogrammes) divided by height (meters) squared, and then categorized according to the World Health Organization criteria: underweight <18.5, normal weight 18.5–24.9, overweight 25–29.9 and obesity ≥30.0 kg/m^2^.^27^ Medical history of diseases was collected from a self‐reported baseline assessment questionnaire. These data were used to create a multimorbidity variable based on the count of diseases medically diagnosed for each participant.^28^

#### Lifestyle behaviours

Self‐reported walking pace was categorized as slow, average or brisk pace. Sleep duration was self‐reported and categorized as short (<7 h/day), normal (7–8 h/day), and long (>9 h/day) sleep. TV viewing was self‐reported and categorized as ≤4 and >4 h/day. Smoking and alcohol status were self‐reported. Smoking was categorized into ‘current’, ‘former’ or ‘never’, and alcohol intake was categorized into not heavy drinker (less than once/two times a week) and heavy drinker (three/four times a week or more). Dietary information was self‐reported using a touch screen questionnaire. Participants were asked how many portions of specified foods they generally ate including: fruit and vegetables, red meat, processed meat, and oily fish. Further details of these measurements can be found in the UK Biobank online protocol (http://www.ukbiobank.ac.uk).

#### Ethical approval

The UK Biobank study was approved by the North West Multi‐Centre Research Ethics Committee (Ref 11/NW/0382 on 17 June 2011), and all participants provided written informed consent to participate in the UK Biobank study. The study protocol is available online (http://www.ukbiobank.ac.uk/). This research was conducted using the UK Biobank resource under application number 7155.

#### Patient involvement

No patients were involved in setting the research question or the outcome measures nor were they involved in developing plans for design or implementation of the study. No patients were asked to advice on interpretation or writing up of results. There are no plans to disseminate the results of the research to study participants.

### Statistical analyses

Baseline characteristics of the study participants by sex‐specific and age‐specific quintiles of handgrip strength are presented as mean ± SD or percentages, as appropriate. We investigated the association between handgrip strength and dementia incidence and mortality using Cox proportional hazard models and time of follow‐up as the time‐dependent variable. A landmark analysis was conducted to reduce the potential for reverse causality, with follow‐up starting 2 years after recruitment. We excluded from the analysis participants with self‐reported diagnosed dementia, Alzheimer's disease and cognitive impairment at baseline as well as those diagnosed in the first 2 years of follow‐up. In addition, we conducted a sensitivity analysis applying a 4‐year landmark analysis.

Non‐linear associations between handgrip strength and dementia outcomes were visually explored using multivariable penalized cubic splines in Cox‐proportional hazard models.^29^ Penalized spline is a technique that balances data fit and smoothness.^30^ Spline curvature is penalized by the integrated second derivative. Knots were selected based on generalized cross validation and were equally spaced across the range of the exposure variable. Spline values were restricted to be linear below the first, and beyond the final, knot to ensure numerical stability.^31^ The results are reported as hazard ratios (HRs) together with 95% confidence intervals (CIs).

The associations between handgrip strength (expressed per 5 kg lower grip strength and by age‐ and sex‐specific quintile) and all‐cause dementia outcomes were presented as HRs and their 95% CIs, obtained from the Cox proportional hazards regression model. Participants in the fifth quintile (highest grip strength) were used as the reference group. In addition, we examined the associations of handgrip strength with incidence and mortality from Alzheimer's and vascular dementia.

We ran three incremental models: Model 1 was adjusted for sociodemographic factors (age, sex, ethnicity, and deprivation index); Model 2 was additionally adjusted for health‐related factors including body mass index categories, multimorbidity (prevalent diabetes, hypertension, CVD, and cancer) as well as long‐standing illness; and Model 3 was additionally adjusted for lifestyle factors including walking pace, sleep duration, TV viewing, smoking, and diet (alcohol, fruit and vegetables, red meat, processed meat, and oily fish intake).

We calculated rate advancement periods (RAPs) to estimate the number of additional chronologic years that would be required to yield the equivalent risk rate estimates of all‐cause dementia incidence or mortality among individuals in the highest quintile for handgrip strength compare to the lowest quintile.^32^ To quantify the potential impact of improving grip strength, we estimated population attributable fractions (PAFs) and potential impact fractions (PIFs) using the standard formulae. PAF were estimated based on two scenarios: Scenario 1 indicates the proportion of all‐cause dementia incidence and mortality attributable to having the lowest grip strength levels; Scenario 2 indicates the dementia cases and deaths attributable to having muscle weakness based on Fried's criteria.^33^ Our PIF estimations considered the following scenario, if participants in Quintiles 1 to 4 improve their grip strength by 1‐quintile. PIFs, in this case, indicate the proportional reduction in dementia cases and deaths participants achieve this scenario.

We also examined the moderation effects of sociodemographic factors (sex, age, and deprivation index), health‐related factors (BMI categories, multimorbidity, and long‐standing illness), and lifestyle behaviours (smoking status, alcohol intake, walking pace, TV viewing, and sleep time) in this association. We fitted interaction terms between handgrip strength (expressed per 5 kg lower handgrip strength) and each of these factors using the fully adjusted model. Then, we stratified the analyses and calculated HRs per 5 kg lower handgrip strength for each of the moderators, separately. In addition, we repeated the moderation analyses using different subgroup categories. Statistical significance was set at two‐sided *P* < 0.05. Analyses were performed using STATA v15 statistical software.

## Results

The median follow‐up period was 9.1 years (inter‐quartile range 8.3 to 9.7) for all‐cause dementia incidence, and 9.3 years (inter‐quartile range 8.7–10.0) for all‐cause dementia mortality. Over the follow‐up period, a total of 4087 people developed dementia, and 1309 died from dementia.

*Table* 1 shows the baseline characteristics of the cohort by sex‐specific and age‐specific quintiles of handgrip strength. In summary, participants in the lowest fifth of handgrip strength were more likely to be deprived, had a higher prevalence of obesity, multimorbidity, smoked, walked more slowly, and spent more time watching TV compared with the highest fifth of handgrip strength.

**Table 1:** Baseline characteristics of the cohort by sex‐and age‐specific quintiles of handgrip strength

	Handgrip strength^a^					
Characteristics	All	Q5 (highest)	Q4	Q3	Q2	Q1 (lowest)
*N*	466 788	90 148	87 841	92 964	99 121	96 714
*Sociodemographics*						
Women	254 535 (54.5)	48 188 (53.5)	46 429 (52.9)	51 735 (55.7)	57 093 (57.6)	51 090 (52.8)
Age, years, mean (SD)	56.51 (8.08)	56.51 (8.08)	56.04 (8.18)	56.99 (8.02)	56.75 (7.95)	57.03 (7.80)
Deprivation index						
Lower (least deprived)	159 290 (34.1)	33 955 (37.7)	31 887 (36.3)	32 748 (35.2)	33 031 (33.3)	27 669 (28.6)
Middle	157 162 (33.7)	30 844 (34.2)	30 158 (34.3)	31 909 (34.3)	33 303 (33.6)	30 948 (32.0)
Higher (most deprived)	150 336 (32.2)	25 349 (28.1)	25 796 (29.4)	28 307 (30.5)	32 787 (33.1)	38 097 (39.4)
Ethnicity						
White	444 023 (95.1)	86 970 (96.5)	84 717 (96.4)	89 289 (96.1)	94 205 (95.0)	88 842 (91.9)
Mixed background	6456 (1.4)	1019 (1.1)	1045 (1.2)	1116 (1.2)	1430 (1.4)	1846 (1.9)
South Asian	8271 (1.8)	409 (0.5)	723 (0.8)	1134 (1.2)	1902 (1.9)	4103 (4.2)
Black	6674 (1.4)	1624 (1.8)	1158 (1.3)	1158 (1.3)	1254 (1.3)	1480 (1.5)
Chinese	1364 (0.3)	126 (0.1)	198 (0.2)	267 (0.3)	330 (0.3)	443 (0.5)
Health‐related factors						
Handgrip strength, kg, mean (SD)	30.73 (10.99)	40.82 (10.64)	35.05 (8.87)	30.83 (8.20)	27.00 (7.73)	21.12 (7.85)
BMI categories						
Underweight	2404 (0.5)	241 (0.3)	325 (0.4)	493 (0.5)	613 (0.6)	732 (0.8)
Normal weight	153823 (33.0)	27 465 (30.5)	29 378 (33.5)	32 011 (34.4)	34 400 (34.7)	30 569 (31.6)
Overweight	198 786 (42.6)	40 003 (44.4)	38 414 (43.7)	39 748 (42.8)	41 189 (41.6)	39 432 (4.8)
Obesity	111 775 (24.0)	22 439 (24.9)	19 724 (22.5)	20 712 (22.3)	22 919 (23.1)	25 981 (26.9)
Multimorbidity						
0 morbidities	164 202 (35.2)	35 643 (39.5)	33 742 (38.4)	33 377 (35.9)	33 962 (34.26)	27 478 (28.4)
1 or more morbidities	302 586 (64.8)	54 505 (60.5)	54 099 (61.6)	59 587 (64.1)	65 159 (65.7)	69 236 (71.6)
Long‐standing illness						
No	317 963 (68.1)	66 904 (74.2)	63 698 (72.5)	65 513 (70.5)	67 045 (67.64)	54 803 (56.7)
Yes	148 825 (31.9)	23 244 (25.8)	24 143 (27.5)	27 451 (29.53)	32 076 (32.4)	41 911 (43.3)
Lifestyle behaviours						
Smoking status						
Never	256 879 (55.0)	48 827 (54.2)	47 887 (54.5)	51 123 (55.0)	55 362 (55.9)	53 680 (55.5)
Former	162 225 (34.8)	32 446 (36.0)	31 107 (35.4)	32 700 (35.2)	33 801 (34.1)	32 171 (33.3)
Current	47 684 V(10.2)	8875 (9.8)	8847 (10.1)	9141 (9.8)	9958 (10.1)	10 863 (11.2)
Alcohol intake						
Daily or almost daily	96 567 (20.7)	20 509 (22.1)	19 405 (22.1)	19 816 (21.3)	19 680 (19.9)	17 157 (17.7)
Three or four times a week	109 762 (23.5)	22 807 (25.1)	22 071 (25.1)	22 449 (24.2)	22 844 (23.1)	19 591 (20.3)
Once or twice a week	120 957 (25.9)	23 368 (25.9)	23 000 (26.2)	24 090 (25.9)	25 896 (26.1)	24 603 (25.4)
One to three times a month	51 966 (11.1)	9924 (11.0)	9486 (10.8)	10 299 (11.1)	11 316 (11.4)	10 941 (11.3)
Special occasions only	52 078 (11.2)	8504 (9.4)	8648 (9.8)	9983 (10.7)	11 572 (11.7)	13 371 (13.8)
Never	35 458 (7.6)	5036 (5.6)	5231 (6.0)	6327 (6.8)	7813 (7.9)	11 051 (11.4)
Walking pace						
Slow pace	36 115 (7.7)	3 560 (4.0)	4278 (4.9)	5526 (5.9)	7650 (7.7)	15 101 (15.6)
Average pace	245 255 (52.5)	44 096 (48.9)	44 562 (50.7)	49 297 (53.0)	54 315 (54.8)	52 985 (54.8)
Brisk pace	185 418 (39.7)	42 492 (47.1)	39 001 (44.4)	38 141 (41.0)	37 156 (37.5)	28 628 (2)
TV viewing, h/days, mean (SD)	2.78 (1.58)	2.62 (1.46)	2.69 (1.51)	2.76 (1.54)	2.82 (1.59)	3.00 (1.75)
Sleep time, h/day						
Short sleep (<7 h/day)	345 140 (73.9)	68 566 (76.1)	66 497 (75.7)	69 719 (75.0)	72 883 (73.5)	67 475 (69.8)
Normal (7–9 h/day)	113 707 (24.4)	20 585 (22.8)	20 180 (23.0)	21 886 (23.5)	24 429 (24.7)	26 627 (27.5)
Long sleep (>9 h/day)	7941 (1.7)	997 (1.1)	1164 (1.3)	1359 (1.5)	1809 (1.8)	2612 (2.7)
Fruit and vegetables intake, g/day	330.61 (193.78)	336.31 (189.96)	331.77(191.30)	332.18 (191.17)	328.44 (190.82)	324.95 (204.58)
Processed meat intake						
Never	43 390 (9.3)	7612 (8.44)	7864 (9.0)	8558 (9.2)	9727 (9.8)	9629 (10.0)
Less than once a week	143 044 (30.6)	28 500 (31.6)	271 162 (30.9)	29 147 (31.4)	30 487 (30.8)	27 748 (28.7)
Once a week	136 570 (29.3)	26 607 (29.5)	26 122 (29.7)	27 076 (29.1)	28 716 (29.0)	28 049 (29.0)
2–4 times a week	125 848 (27.0)	24 206(26.9)	23 359 (26.6)	24 851 (26.7)	26 479 (26.7)	26 953 (27.9)
5–6 times a week	14 321 (3.07)	2609 (2.9)	2698 (3.1)	2672 (2.9)	2951 (3.0)	3391 (3.5)
Once or more daily	3615 (0.8)	614 (0.7)	636 (0.7)	660 (0.7)	761 (0)	
Red meat intake, portion/day, mean (SD)	2.11 (1.43)	2.15(1.42)	2.16 (1.41)	2.10 (1.40)	2.08 (1.43)	2.10 (1.51)
Oily fish intake						
Never	50135 (10.7)	8015 (8.9)	8548 (9.7)	9404 (10.1)	11 082 (11.2)	13 086 (13.5)
Less than once a week	155 042 (33.2)	30 228 (33.5)	29 506 (33.6)	30 833 (33.2)	32 796 (33.1)	31 679 (32.8)
Once a week	177 513 (38.0)	35 071 (38.9)	33 922 (38.6)	35 812 (38.5)	37 558 (37.9)	35 150 (36.3)
2–4 times a week	79 767 (17.1)	15 978 (17.7)	15 038 (17.12)	16 088 (17.3)	16 837 (17.0)	15 826 (16.7)
5–6 times a week	3288 (0.7)	671 (0.7)	633 (0.7)	634 (0.7)	627 (0.6)	723 (0.8)
Once or more daily	1043 (0.2)	185 (0.2)	194 (0.2)	193 (0.2)	221 (0.2)	250 (0.3)

Values are percentage, unless otherwise stated.

^a^ Sex‐specific and age‐specific quintiles of handgrip strength.

**Figure 1:**
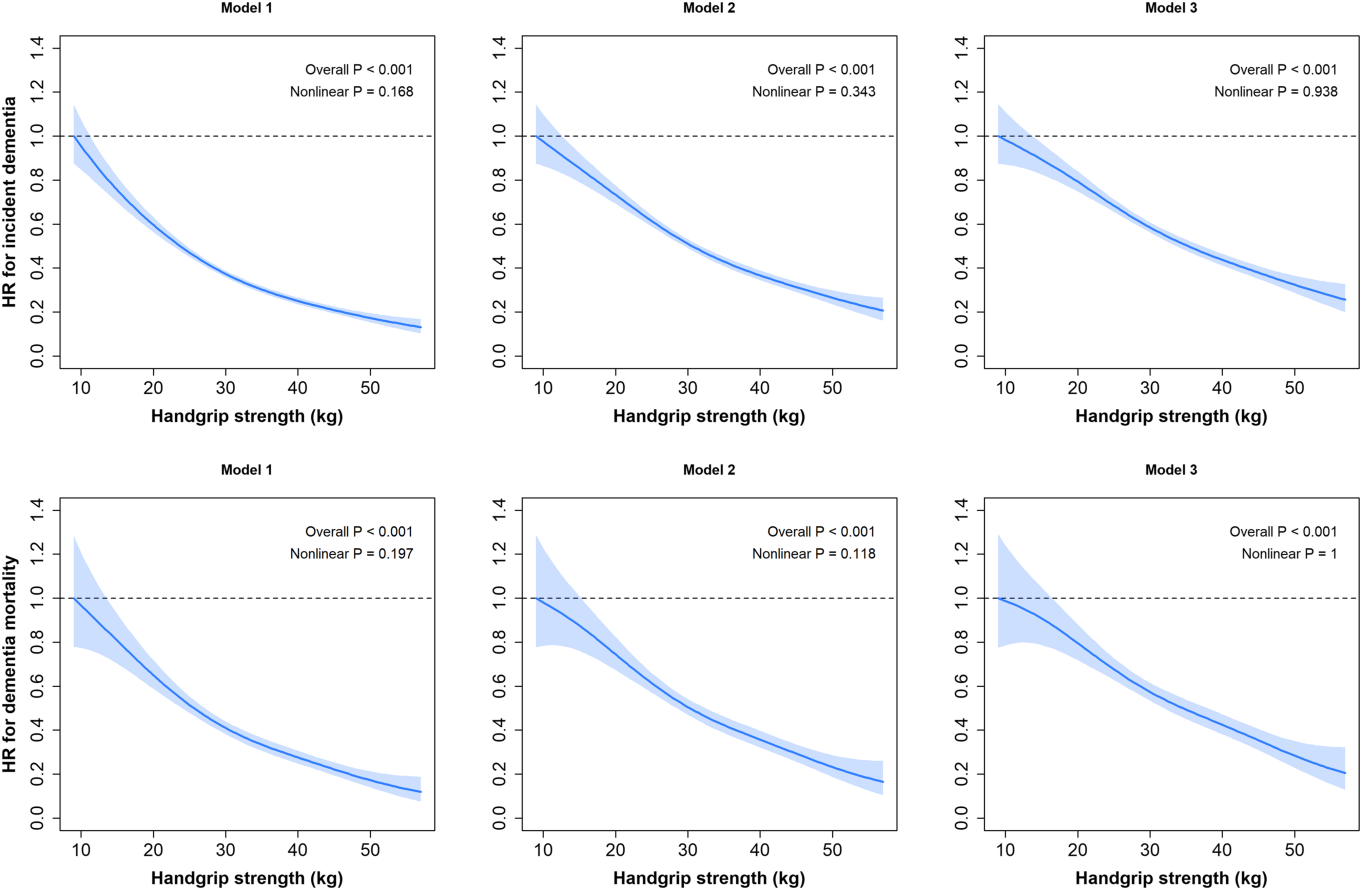
Association of handgrip strength with all‐cause dementia incidence (top graphs) and mortality (bottom graphs). Data are presented as hazard ratios (HRs) and 95% confidence interval (CI). Handgrip strength was expressed in absolute terms. All analyses were conducted using a 2 years landmark. Model 1 was adjusted for age, sex, ethnicity, and deprivation index. Model 2 was additionally adjusted for health‐related factors including body mass index categories, multimorbidity (prevalent diabetes, hypertension, cardiovascular disease, and cancer), and long‐standing illness. Model 3 was additionally adjusted by lifestyle behaviours including walking pace, sleep duration, watching TV, smoking, and dietary intake (alcohol, fruits and vegetables, red meat, processed meat, and oily fish intake).

As shown in *Figure* 1, there was no evidence that the association between handgrip strength and all‐cause dementia incidence was non‐linear (*P* non‐linear >0.05). For the minimally adjusted model, the risk of developing dementia was 22% higher per 5 kg lower grip strength (*Table* 2). When age‐sex‐specific quintiles of grip strength were fitted into the model, those in the lowest quintile had a higher risk of incident dementia (HR: 2.17 [95% CI: 1.96; 2.41]) compared to those in the highest fifth. On average, in each quintile, lower grip strength was associated with a 21% higher risk of developing dementia (*Table* 2). When the analyses were fully adjusted for sociodemographic, health, and lifestyle‐related covariates, the associations remained significant, but the magnitudes were slighted attenuated (HR: 1.14 [95% CI: 1.12; 1.17] per 5 kg lower grip and HR: 1.15 [95% CI: 1.12; 1.17] per quintile lower grip strength) (*Table* 2). Similar patterns were observed when analyses were performed using a 4 year landmark (Supporting information *Figure* S1). Similar associations were found for the associations of handgrip strength with incidence from Alzheimer's and vascular dementia (*Table* S1).

**Table 2:** Associations of handgrip strength with incidence and mortality from all‐cause dementia

	All‐cause dementia incidence			
		Model 1	Model 2	Model 3
Quintiles of grip strength	*n*/events	HR (95% CI)	HR (95% CI)	HR (95% CI)
Q5 (highest)	90 145/487	Ref.	Ref.	Ref.
Q4	87 830/572	1.17 (1.04; 1.33)	1.15 (1.02; 1.30)	1.13 (1.00;1.27)
Q3	92 957/792	1.37 (1.23; 1.54)	1.32 (1.18; 1.48)	1.29 (1.15; 1.45)
Q2	99 096/948	1.61 (1.44; 1.80)	1.50 (1.35; 1.67)	1.45 (1.30; 1.62)
Q1 (lowest)	96 679/1288	2.17 (1.96; 2.41)	1.84 (1.66; 2.05)	1.72 (1.55; 1.92)
Quintile change	466 707/4087	1.21 (1.17; 1.24)	1.16 (1.14; 1.19)	1.15 (1.12; 1.17)
*P* for trend		<0.001	<0.001	<0.001
HR per 5 kg lower handgrip strength	466 707/4087	1.22 (1.20‐1.25)	1.16 (1.13‐1.19)	1.14 (1.12‐1.17)

	All‐cause dementia mortality			
		Model 1	Model 2	Model 3
Quintiles of handgrip	*n*/events	HR (95% CI)	HR (95% CI)	HR (95% CI)
Q5 (highest)	90 148/160	Ref.	Ref.	Ref.
Q4	87 841/207	1.35 (1.10; 1.66)	1.32 (1.07; 1.62)	1.29 (1.05; 1.58)
Q3	92 964/253	1.42 (1.16; 1.73)	1.36 (1.12; 1.66)	1.32 (1.08; 1.61)
Q2	99 121/297	1.68 (1.38; 2.03)	1.58 (1.30; 1.91)	1.52 (1.25; 1.84)
Q1 (lowest)	96 714/392	2.27 (1.89; 2.73)	2.02 (1.67; 2.43)	1.87 (1.55; 2.26)
Quintile change	466 788/1309	1.21 (1.16; 1.26)	1.17 (1.13; 1.22)	1.15 (1.07; 1.20)
*P* for trend		<0.001	<0.001	<0.001
HR per 5 kg lower handgrip strength	466 788/1309	1.23 (1.18‐1.28)	1.19 (1.14‐1.23)	1.17 (1.12‐1.21)

Data presented as hazard ratio [HR, 95% confidence interval (CI)]. All analyses were conducted using a 2 years landmark. Model 1 was adjusted for age, sex, ethnicity, and deprivation index. Model 2 was additionally adjusted for health‐related factors including body mass index categories, multimorbidity (prevalent diabetes, hypertension, CVD, and cancer), and long‐standing illness. Model 3 was additionally adjusted by lifestyle behaviours including walking pace, sleep duration, watching TV, smoking, and dietary intake (alcohol, fruits and vegetables, red meat, processed meat, and oily fish intake).

For all‐cause dementia mortality, there was evidence of a non‐linear association between handgrip strength and dementia in the minimally adjusted models; however, in the maximally adjusted model (Model 3), the association became linear (*Figure* 1). For the minimally adjusted model, a 5 kg lower grip strength was associated with a 23% higher risk of dementia mortality (*Table* 2). When grip strength was expressed in quintiles, those in the lowest quintile had a higher risk of dying from dementia compared with the highest quintile (HR: 2.27 [95% CI: 1.89; 2.73]). On average, the risk of dementia mortality increased by 21% per each quintile lower of grip strength (*Table* 2). When the analyses were adjusted for health and lifestyle factors, including BMI (Model 3) the associations were slightly attenuated but remained significant (*Table* 2). Similar patterns were observed when the analyses were conducted with a 4‐year landmark (*Figure* S1). Similar associations were found for the associations of handgrip strength with mortality from Alzheimer's and vascular dementia (*Table* S1).

When the associations of a 5 kg lower grip strength and dementia incidence and mortality were stratified by sociodemographic, lifestyle, and health‐related factors no significant interactions were found (*Figure* S2 and S3). When the moderators were further stratified for BMI, smoking, walking pace, and sleep time, the findings were consistent (*Tables* S2 and S3).

The RAP analysis revealed that individuals with the lowest grip strength (Quintile 1) will experience the same dementia incidence and mortality rate as those among the highest fifth for grip strength who were 3.0 years (95% CI: 2.47; 3.40) and 2.7 (95% CI: 2.02; 3.32) years older, respectively. In addition, the PAF analysis showed that muscle weakness based on the Fried criteria, if causal, accounted for 10.0% of incident all‐cause dementia and 10.4% of all‐cause dementia mortality (*Table* 3). Based on age‐specific and sex‐specific quintiles, being in the lowest fifth for grip strength accounted for 30.1% and 32.3% of all dementia incidence cases and deaths, respectively. Further, if all individuals in Quintiles 1 to 4 of grip strength improved their strength by 1‐quintile, 11.5% of dementia cases and 12.2% of dementia deaths could have been prevented (*Table* 3)

**Table 3:** Population attributable fractions and potential impact fractions

	Dementia incidence	Dementia mortality
PAF (95% CI)	30.11 (25.28; 34.62)	32.26 (24.70; 39.71)
Attributable to the lowest quintile of grip strength		
Attributable to muscle weakness based on Fried's criteria	9.98 (8.10; 11.83)	10.41 (7.34; 13.39)
PIF (95% CI)		
If those in Q1–Q4 improved their grip strength for 1‐quintile	11.50 (7.69; 16.52)	12.19 (7.52; 16.58)

Estimated based on RR shown in Table 2

## Discussion

The main finding of the present study was that lower handgrip strength was associated with a higher risk of all‐cause dementia incidence and mortality, independent of a wide range of confounding factors. In addition, these findings were consistent across sociodemographic, health‐related, and lifestyle behavioural subgroups. Our findings may have important clinical implications for the identification of high‐risk individuals as handgrip strength is easily measured, cheap, and highly reproducible in clinical practice.^34^ As such, handgrip strength may be a useful method of identifying people with muscle weakness who are at high risk of all‐cause dementia and who might benefit from further neurodegenerative health assessments. However, future studies assessing the feasibility and prediction ability of using handgrip strength as a screening tool are needed.

Our study extends the limited evidence to date regarding the association between handgrip strength and dementia risk. Previous studies have been conducted mainly in older adults based on relatively small cohorts that included individuals with major illness such cognitive impairment.^4^, ^5^, ^6^ A previous study in 2288 older adults (mean age 76 years) found that 1‐point higher handgrip strength was associated with a 13% lower dementia incidence.^6^ While this study controlled for sociodemographic and health‐related variables, the analyses lacked adjustment for some relevant confounding lifestyle behavioural factors such as dietary intake, physical activity, smoking, and alcohol status.^6^ Another study among Catholic clergy members (877 participants, mean age 76 years) identified that, after adjusting for a set of sociodemographic and lifestyle factors, a 0.5 kg lower handgrip strength was associated with a 9% higher risk of Alzheimer's disease (HR, 0.91 [95% CI: 0.88; 0.94]). However, these findings were based on a selected cohort that differed in important ways (i.e. education, socioeconomic status, and lifestyle) from older adults in the general population.^5^ In another study of 970 community‐based older persons (729 women, mean age 80 years, SD = 7), where a muscle strength score was derived from nine muscle groups, 1‐point higher strength was associated with a 43% lower risk of Alzheimer's disease (HR: 0.57 [95% CI: 0.41; 0.79]), even after adjustment for BMI, physical activity, pulmonary function, CVD, and apolipoprotein e4 status.^4^ They also reported that muscle strength was associated with a decreased risk of mild cognitive impairment, a precursor to Alzheimer's disease (HR: 0.67 [95%CI: 0.54; 0.84]).^4^ However, our study extends limited evidence on the association of handgrip strength with dementia risk and mortality beyond older and frail adults. Specifically, we found that, in the fully adjusted model, for each 5 kg decrement in handgrip strength, there was a 14% higher risk of all‐cause dementia and a 17% higher risk of dementia death. Indeed, those in the lowest quintile had 72% higher risk of developing dementia and 87% higher risk of dying from dementia compared with those in the highest quintile. To note, the risk was similar among the most prevalent type of dementia; for example, those in the lowest quintile had 81% vs. 88% higher risk of developing Alzheimer's vs. vascular dementia, and had 82% vs. 98% higher risk of dying from Alzheimer's vs. vascular dementia compared with those in the highest quintile. The latter highlights that efforts should focus on those with very low muscular strength. These associations were independent of age, sex, ethnicity, deprivation index, body mass index, multimorbidity, long‐standing illness, and other confounding behavioural factors including walking pace, sleep duration, watching TV, smoking, and alcohol status, and dietary patterns. In addition, there was no suggestion of moderation on the basis of subgroups of sociodemographic, health‐related, and lifestyle behaviour factors. We have extended previous studies not only by examining for the first time the association with all‐cause dementia mortality and the most prevalent subtypes (Alzheimer's and vascular dementia), but also by studying a large prospective cohort that includes middle‐aged adults, by adjusting for a wide range of potential confounding factors, and by assessing whether the association between grip strength and dementia differed by subgroups of the population.

Our RAPs analyses highlighted the clinical implications of the study by showing that, individuals in the lowest fifth of grip strength reached the same risk rate of developing and dying from dementia 3.0 and 2.7 years earlier than those in the highest fifth for strength, respectively. In addition, the PAFs suggested that 10.0% of the dementia cases and 10.4% of the dementia deaths are attributable to muscle weakness based on Fried's criteria.^33^, ^35^ However, 30.1% of dementia cases and 32.3% of dementia deaths are attributable to being in the lowest quintile of grip strength. We have also estimated that 11.5% and 12.2% of dementia cases and deaths, respectively, could have been prevented, if we were to assume causality, if participants in Quintiles 1 to 4 of grip strength improve their strength levels by 1‐quintile, which a feasible scenario if the right interventions are implemented.^36^, ^37^, ^38^

### Strengths and limitations

This study has several strengths. First, the UK Biobank is relatively representative of the general population in terms of age and sex, but is only partially representative in terms of lifestyle, as is generally healthier than the wider UK population. Second, a wide range of sociodemographic, health, and behavioural confounders were controlled for in our analyses, including removal of all participants with all‐cause dementia at baseline. Third, our study had sufficient power to allow subgroup analyses by sociodemographic, health‐related, and lifestyle behaviour factors. Lastly, handgrip strength was assessed using validated methods, trained staff, and standard operating procedures,^25^ and dementia cases were identified using routinely collected hospital admission records. However, several limitations must be acknowledged. Reverse causality is possible in any observational study; although we attempted to minimize this risk by performing landmark analysis of events occurring from 2 years after recruitment in our main analysis, and 4 years after recruitment in a sensitivity analysis. Future studies with longer follow‐up should apply more conservative landmark analyses. In addition, dementia mortality may be under recorded from deaths registers as has been suggested elsewhere.^39^ However, a previous study using UK Biobank data showed high positive predictive values for all‐cause dementia incidence^21^; it is uncertain how a degree of underdetection could have influenced our results, although we speculate that this may have produced more conservative hazard ratios for dementia risk. Although our PAF suggest that theatrically muscle weakness account for an important proportion on dementia cases and deaths, the interpretation should be taken with caution as our study cannot infer causality. Similarly, although we adjusted for major confounding factors, residual confounding from unknown or unmeasured factors still remains possible. Effect sizes may be smaller than some previous ageing studies because our population was relatively young for the expression of late‐life cognitive impairment. However, the age range of the cohort, covering mid to late adulthood, also enabled us to consider both earlier‐onset and later‐onset dementias.

## Conclusion

In conclusion, lower handgrip strength was associated with a higher risk of all‐cause dementia incidence and mortality, independent of a wide range of sociodemographic, health, and behavioural confounders, and was consistent across subgroups of participants. These findings could have important clinical and public health implications, as handgrip strength is a quick and reproducible measurement that could be used in clinical practice for identifying persons at risk of the earliest manifestation of dementia, and who may benefit most from intervention if causality is established.

## Funding

This study was funded by by the Wellcome Trust, Medical Research Council, Department of Health, Scottish government, and Northwest Regional Development Agency. It has also had funding from the Welsh assembly government and the British Heart Foundation. I.E.C. is supported by the Spanish Ministry of Economy and Competitiveness (RTI2018‐095284‐J‐100) and the Spanish Ministry of Science and Innovation (RYC2019‐027287‐I). I.E.C. and F.B.O. are supported by the University of Granada, *Plan Propio de Investigación* 2016, Excellence actions: Units of Excellence; Scientific Excellence Unit on Exercise and Health (UCEES).

## Conflict of interest

All authors have completed the ICMJE uniform disclosure form online (www.icmje.org/coi_disclosure.pdf) and declare: UK Biobank was established by the Wellcome Trust medical charity, Medical Research Council, Department of Health, Scottish government, and Northwest Regional Development Agency; no financial relationships with any organizations that might have an interest in the submitted work in the previous 3 years; no other relationships or activities that could appear to have influenced the submitted work. Irene Esteban‐Cornejo declares that she has no conflict of interest. Frederick Ho declares that he has no conflict of interest. Fanny Petermann‐Rocha declares that she has no conflict of interest. Donald M. Lyall declares that he has no conflict of interest. David Martinez‐Gomez declares that he has no conflict of interest. Veronica Cabanas‐Sanchez declares that she has no conflict of interest. Francisco B. Ortega declares that he has no conflict of interest. Charles H. Hillman declares that he has no conflict of interest. Jason M. R. Gill declares that he has no conflict of interest. Terrance J. Quinn declares that she has no conflict of interest. Naveed Sattar declares that he has no conflict of interest. Jill P. Pell declares that she has no conflict of interest. Stuart R. Gray declares that he has no conflict of interest. Carlos Celis‐Morales declares that he has no conflict of interest.

## Supporting information


**Table S1.** Associations of handgrip strength with incidence and mortality from Alzheimer's and Vascular Dementia.
**Table S2.** Hazard ratio (95% CI) for all‐cause dementia incidence per 5 kg lower handgrip strength stratified by subgroups.
**Table S3.** Hazard ratio (95% CI) for all‐cause dementia mortality per 5 kg lower handgrip strength stratified by subgroups.
**Figure S1.** Association of handgrip strength with all‐cause dementia incidence (top graphs) and mortality (bottom graphs) with a 4‐years landmark analyses.
Data is presented as hazard ratios and their 95% CI. Handgrip strength was expressed in absolute terms. Model 1 was adjusted for age, sex, ethnicity and deprivation index. Model 2 was additionally adjusted for health‐related factors including body mass index categories, multimorbidity (prevalent diabetes, hypertension, CVD and cancer) as well as long‐standing illness. Model 3 was additionally adjusted by lifestyle behaviours including walking pace, sleep duration, watching TV, smoking and dietary intake (alcohol, fruits and vegetables, red meat, processed meat and oily fish intake).
**Figure S2.** Association between 5‐kg lower handgrip strength and incident dementia by sociodemographic, health‐related and lifestyle factors.
Data is presented as hazard ratios and 95% CI. All analyses were conducted using a 2‐years landmark. Model was adjusted for age, sex, ethnicity, deprivation index, body mass index categories, multimorbidity (prevalent diabetes, hypertension, CVD and cancer) as well as long‐standing illness, walking pace, sleep duration, watching TV, smoking and dietary intake (alcohol, fruits and vegetables, red meat, processed meat and oily fish intake), excluding the appropriated grouping variable. Non‐heavy intake was defined as drinking alcohol less than twice or one time a week; normal sleep was defined as sleeping 7‐9 hour/day.
**Figure S3.** Association between 5‐kg lower handgrip strength and dementia mortality by sociodemographic, health‐related and lifestyle factors.
Data is presented as hazard ratios and 95% CI. All analyses were conducted using a 2‐years landmark. Model was adjusted for age, sex, ethnicity, deprivation index, body mass index categories, multimorbidity (prevalent diabetes, hypertension, CVD and cancer) as well as long‐standing illness, walking pace, sleep duration, watching TV, smoking and dietary intake (alcohol, fruits and vegetables, red meat, processed meat and oily fish intake), excluding the appropriated grouping variable. Non‐heavy intake was defined as drinking alcohol less than twice or one time a week; normal sleep was defined as sleeping 7‐9 hour/day.
