# Supplementary material for: Handgrip strength and all‐cause dementia incidence and mortality: findings from the UK Biobank prospective cohort study
Source: J Cachexia Sarcopenia Muscle. 2022 Apr 21;13(3):1514–25. doi: 10.1002/jcsm.12857 (PMC9178163; doi:10.1002/jcsm.12857)
Supplement: Supplementary file 1 — Table S1. Associations of handgrip strength with incidence and mortality from Alzheimer's and Vascular Dementia. Table S2. Hazard ratio (95% CI) for all‐cause dementia incidence per 5 kg lower handgrip strength stratified by subgroups. Table S3. Hazard ratio (95% CI) for all‐cause dementia mortality per 5 kg lower handgrip strength stratified by subgroups. Figure S1. Association of handgrip strength with all‐cause dementia incidence (top graphs) and mortality (bottom graphs) with a 4‐years landmark analyses. Data is presented as hazard ratios and their 95% CI. Handgrip strength was expressed in absolute terms. Model 1 was adjusted for age, sex, ethnicity and deprivation index. Model 2 was additionally adjusted for health‐related factors including body mass index categories, multimorbidity (prevalent diabetes, hypertension, CVD and cancer) as well as long‐standing illness. Model 3 was additionally adjusted by lifestyle behaviours including walking pace, sleep duration, watching TV, smoking and dietary intake (alcohol, fruits and vegetables, red meat, processed meat and oily fish intake). Figure S2. Association between 5‐kg lower handgrip strength and incident dementia by sociodemographic, health‐related and lifestyle factors. Data is presented as hazard ratios and 95% CI. All analyses were conducted using a 2‐years landmark. Model was adjusted for age, sex, ethnicity, deprivation index, body mass index categories, multimorbidity (prevalent diabetes, hypertension, CVD and cancer) as well as long‐standing illness, walking pace, sleep duration, watching TV, smoking and dietary intake (alcohol, fruits and vegetables, red meat, processed meat and oily fish intake), excluding the appropriated grouping variable. Non‐heavy intake was defined as drinking alcohol less than twice or one time a week; normal sleep was defined as sleeping 7‐9 hour/day. Figure S3. Association between 5‐kg lower handgrip strength and dementia mortality by sociodemographic, health‐related and lifesty [file JCSM-13-1514-s001.pdf]

Supplementary Material

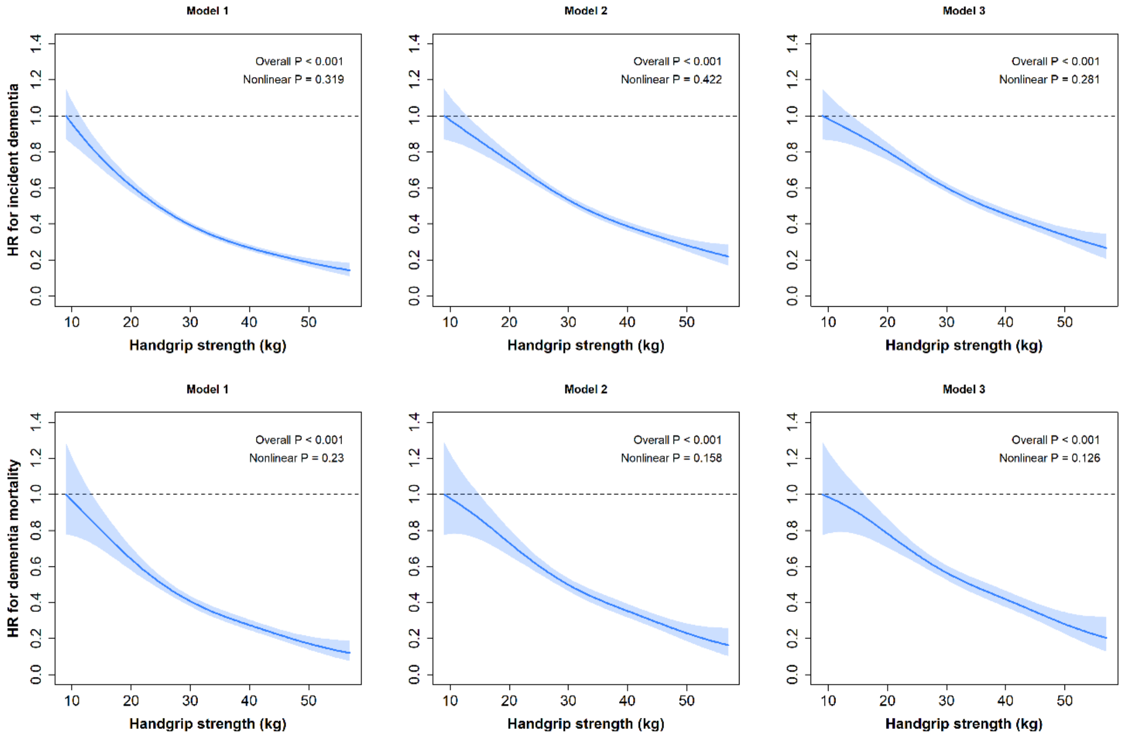

Appendix Figure S1. Association of handgrip strength with all-cause dementia incidence (top graphs) and mortality (bottom graphs) with a 4-years landmark analyses.

Data is presented as hazard ratios and their 95% CI. Handgrip strength was expressed in absolute terms. Model 1 was adjusted for age, sex, ethnicity and deprivation index. Model 2 was additionally adjusted for health-related factors including body mass index categories, multimorbidity (prevalent diabetes, hypertension, CVD and cancer) as well as long-standing illness. Model 3 was additionally adjusted by lifestyle behaviors including walking pace, sleep duration, watching TV, smoking and dietary intake (alcohol, fruits and vegetables, red meat, processed meat and oily fish intake).

Subgroup	Hazard Ratio (95% CI)	P-value	P for interation
**Sex**			
Women	1.12 (1.08-1.17)	<0.001	0.595
Men	1.15 (1.12-1.18)	<0.001	
**Age**			
<65 years	1.25 (1.21-1.28)	<0.001	0.002
≥65 years	1.14 (1.11-1.19)	<0.001	
**Deprivation index**			
Affluent	1.15 (1.11-1.19)	<0.001	0.642
Deprived	1.14 (1.10-1.17)	<0.001	
**Body mass index**			
Normal-weight	1.15 (1.10-1.20)	<0.001	0.720
Overweight/obesity	1.14 (1.11-1.17)	<0.001	
**Multimorbidity**			
None	1.22 (1.15-1.30)	<0.001	0.491
One or more	1.13 (1.11-1.17)	<0001	
**Long-standing illness**			
No	1.16 (1.12-1.21)	<0.001	0.105
Yes	1.13 (1.10-1.16)	<0.001	
**Smoking status**			
Never	1.15 (1.11-1.19)	<0.001	0.996
Former/current	1.14 (1.10-1.17)	<0.001	
**Alcohol intake**			
Non-heavy intake	1.13 (1.10-1.16)	<0.001	0.302
Heavy intake	1.17 (1.13-1.21)	<0.001	
**Walking pace**			
Average/ brisk	1.15 (1.12-1.18)	<0.001	0.253
Slow	1.10 (1.06-1.15)	<0.001	
**TV viewing**			
≤4h/d	1.15 (1.12-1.18)	<0.001	0.450
>4h/d	1.12 (1.06-1.15)	<0.001	
**Sleep time**			
Normal sleep	1.14 (1.11-1.17)	<0.001	0.609
Short/long sleep	1.15 (1.11-1.20)	<0.001	
**All**	1.14 (1.12-1.17)	<0.001	

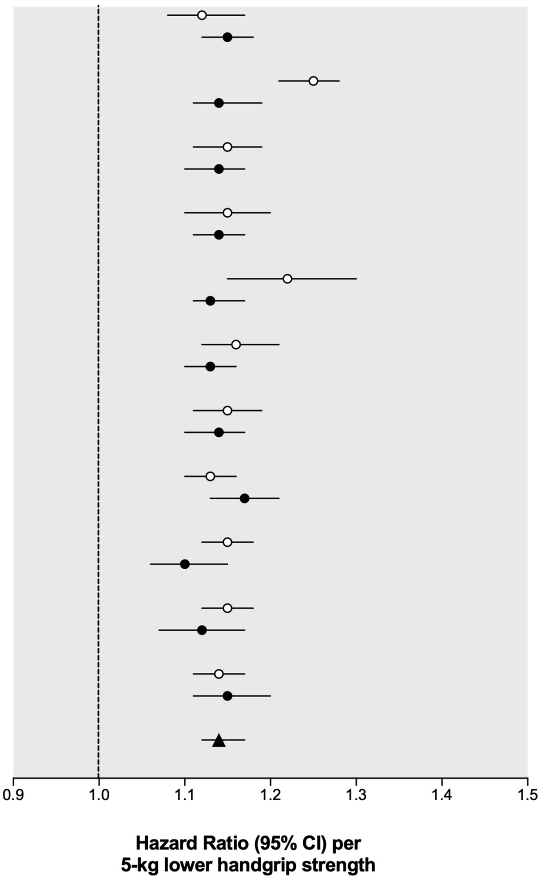

Appendix Figure S2. Association between 5-kg lower handgrip strength and incident dementia by sociodemographic, health-related and lifestyle factors.

Data is presented as hazard ratios and 95% CI. All analyses were conducted using a 2-years landmark. Model was adjusted for age, sex, ethnicity, deprivation index, body mass index categories, multimorbidity (prevalent diabetes, hypertension, CVD and cancer) as well as long-standing illness, walking pace, sleep duration, watching TV, smoking and dietary intake (alcohol, fruits and vegetables, red meat, processed meat and oily fish intake), excluding the appropriated grouping variable. Non-heavy intake was defined as drinking alcohol less than twice or one time a week; normal sleep was defined as sleeping 7-9hour/day.

Subgroup	Hazard Ratio (95% CI)	P-value	P for interation
**Sex**			
Women	1.13 (1.05-1.21)	0.001	0.404
Men	1.18 (1.13-1.23)	<0.001	
**Age**			
<65 years	1.29 (1.22-1.37)	<0.001	0.139
≥65 years	1.16 (1.11-1.22)	<0.001	
**Deprivation index**			
Affluent	1.20 (1.14-1.27)	<0.001	0.217
Deprived	1.13 (1.07-1.19)	<0.001	
**Body mass index**			
Normal-weight	1.14 (1.06-1.22)	<0.001	0.177
Overweight/obesity	1.17 (1.12-1.23)	<0.001	
**Multimorbidity**			
None	1.25 (1.13-1.37)	<0.001	0.983
One or more	1.15 (1.10-1.20)	<0.001	
**Long-standing illness**			
No	1.21 (1.14-1.29)	<0.001	0.443
Yes	1.13 (1.07-1.19)	<0.001	
**Smoking status**			
Never	1.17 (1.10-1.24)	<0.001	0.093
Former/current	1.17 (1.11-1.23)	<0.001	
**Alcohol intake**			
Non-heavy intake	1.13 (1.08-1.19)	<0.001	0.891
Heavy intake	1.22 (1.15-1.29)	<0.001	
**Walking pace**			
Average/ brisk	1.17 (1.11-1.22)	<0.001	0.886
Slow	1.15 (1.06-1.25)	<0.001	
**TV viewing**			
≤4h/d	1.16 (1.11-1.22)	<0.001	0.733
>4h/d	1.18 (1.09-1.27)	<0.001	
**Sleep time**			
Normal sleep	1.16 (1.11-1.22)	<0.001	0.780
Short/long sleep	1.18 (1.10-1.27)	<0.001	
**All**	1.17 (1.12-1.21)	<0.001	

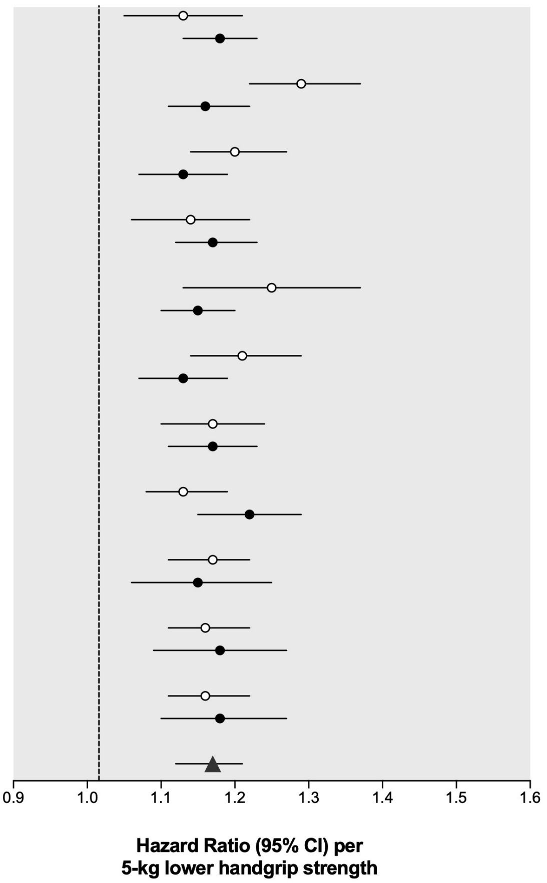

Appendix Figure S3. Association between 5-kg lower handgrip strength and dementia mortality by sociodemographic, health-related and lifestyle factors.

Data is presented as hazard ratios and 95% CI. All analyses were conducted using a 2-years landmark. Model was adjusted for age, sex, ethnicity, deprivation index, body mass index categories, multimorbidity (prevalent diabetes, hypertension, CVD and cancer) as well as long-standing illness, walking pace, sleep duration, watching TV, smoking and dietary intake (alcohol, fruits and vegetables, red meat, processed meat and oily fish intake), excluding the appropriated grouping variable. Non-heavy intake was defined as drinking alcohol less than twice or one time a week; normal sleep was defined as sleeping 7-9hour/day.

Appendix Table S1. Associations of handgrip strength with incidence and mortality from Alzheimer's and Vascular Dementia.

**Alzheimer incidence**				
		Model 1	Model 2	Model 3
Quintiles of grip strength	*n*/events	HR (95% CI)	HR (95% CI)	HR (95% CI)
Q5 (highest)	90145/211	Ref.	Ref.	Ref.
Q4	87830/257	1.22 (1.02; 1.46)	1.20 (1.00; 1.43)	1.18 (0.98;1.42)
Q3	92957/362	1.44 (1.21; 1.70)	1.39 (1.17; 1.64)	1.37 (1.15; 1.62)
Q2	99096/408	1.60 (1.35; 1.88)	1.50 (1.27; 1.78)	1.47 (1.25; 1.74)
Q1 (lowest)	96679/546	2.12 (1.81; 2.49)	1.89 (1.61; 2.22)	1.81 (1.54; 2.13)
Quintile change	466707/1784	1.20 (1.16; 1.24)	1.16 (1.12; 1.20)	1.15 (1.11; 1.19)
P for trend		<0.001	<0.001	<0.001
HR per 5kg lower handgrip strength	466707/1784	1.20 (1.16-1.24)	1.16 (1.12-1.20)	1.14 (1.11-1.18)
**Alzheimer mortality**				
		Model 1	Model 2	Model 3
Quintiles of handgrip	*n*/events	HR (95% CI)	HR (95% CI)	HR (95% CI)
Q5 (highest)	90148/67	Ref.	Ref.	Ref.
Q4	87841/87	1.37 (0.99; 1.88)	1.34 (0.98; 1.85)	1.33 (0.97; 1.83)
Q3	92964/96	1.30 (0.95; 1.77)	1.26 (0.92; 1.72)	1.25 (0.91; 1.71)
Q2	99121/93	1.29 (0.94; 1.76)	1.24 (0.91; 1.70)	1.23 (0.90; 1.67)
Q1 (lowest)	96714/135	1.94 (1.45; 2.61)	1.86 (1.38; 2.50)	1.82 (1.35; 2.46)
Quintile change	466788/478	1.14 (1.07; 1.22)	1.13 (1.06; 1.21)	1.12 (1.05; 1.20)
P for trend		<0.001	<0.001	0.001
HR per 5kg lower handgrip strength	466788/478	1.14 (1.07-1.22)	1.13 (1.06-1.21)	1.12 (1.05-1.20)
**Vascular dementia incidence**				
		Model 1	Model 2	Model 3
Quintiles of grip strength	*n*/events	HR (95% CI)	HR (95% CI)	HR (95% CI)
Q5 (highest)	90145/100	Ref.	Ref.	Ref.
Q4	87830/115	1.15 (0.88; 1.50)	1.12 (0.85; 1.46)	1.09 (0.83; 1.42)
Q3	92957/185	1.56 (1.22; 1.99)	1.49 (1.17; 1.90)	1.44 (1.13; 1.84)
Q2	99096/193	1.59 (1.25; 2.02)	1.44 (1.13; 1.84)	1.37 (1.08; 1.75)
Q1 (lowest)	96679/325	2.64 (2.11; 3.30)	2.09 (1.66; 2.62)	1.88 (1.49; 2.37)
Quintile change	466707/918	1.27 (1.21; 1.34)	1.20 (1.14; 1.26)	1.17 (1.11; 1.23)
P for trend		<0.001	<0.001	<0.001
HR per 5kg lower handgrip strength	466707/918	1.29 (1.23-1.35)	1.20 (1.15-1.25)	1.17 (1.12-1.22)
**Vascular dementia mortality**				
		Model 1	Model 2	Model 3
Quintiles of handgrip	*n*/events	HR (95% CI)	HR (95% CI)	HR (95% CI)
Q5 (highest)	90148/28	Ref.	Ref.	Ref.
Q4	87841/37	1.37 (0.84; 2.25)	1.35 (0.82; 2.20)	1.30 (0.79; 2.12)
Q3	92964/50	1.60 (1.01; 2.55)	1.53 (0.96; 2.44)	1.47 (0.93; 2.34)
Q2	99121/57	1.85 (1.17; 2.91)	1.68 (1.06; 2.64)	1.57 (0.99; 2.48)
Q1 (lowest)	96714/83	2.77 (1.80; 4.26)	2.22 (1.44; 3.44)	1.98 (1.27; 3.08)
Quintile change	466788/255	1.27 (1.16; 1.39)	1.20 (1.09; 1.32)	1.17 (1.06; 1.28)
P for trend		<0.001	<0.001	0.001
HR per 5kg lower handgrip strength	466788/255	1.26 (1.16-1.37)	1.18 (1.08-1.28)	1.14 (1.05-1.24)

Data presented as hazard ratio (HR, 95%CI). All analyses were conducted using a 2-years landmark. Model 1 was adjusted for age, sex, ethnicity and deprivation index. Model 2 was additionally adjusted for health-related factors including body mass index categories, multimorbidity (prevalent diabetes, hypertension, CVD and cancer) as well as long-standing illness. Model 3 was additionally adjusted by lifestyle behaviors including walking pace, sleep duration, watching TV, smoking and dietary intake (alcohol, fruits and vegetables, red meat, processed meat and oily fish intake).

Appendix Table S2. Hazard ratio (95% CI) for all-cause dementia incidence per 5kg lower handgrip strength stratified by subgroups

	N	Events	HR	Lower (95% CI)	Upper (95% CI)	P	P for interaction
Body mass index							
Normal weight	153797	1220	1.15	1.10	1.20	<0.001	0.752
Overweight	198749	1717	1.15	1.11	1.19	<0.001	
Obesity	111758	1119	1.12	1.08	1.17	<0.001	
Smoking status							
Never	256839	1880	1.15	1.11	1.19	<0.001	0.895
Former	162194	1784	1.14	1.10	1.17	<0.001	
Current	47674	423	1.14	1.07	1.22	<0.001	
Walking pace							
Slow pace	36097	792	1.10	1.06	1.15	<0.001	0.284
Average pace	245210	2198	1.13	1.09	1.16	<0.001	
Brisk pace	185400	1097	1.20	1.14	1.25	<0.001	
Sleep time							
Normal sleep (7-9 h/d)	345077	2873	1.14	1.11	1.17	<0.001	0.530
Short sleep (<7 h/d)	113694	1029	1.16	1.11	1.21	<0.001	
Long sleep (>9 h/d)	7936	185	1.12	1.02	1.23	0.022	

Data presented as hazard ratio (HR, 95%CI). All analyses were conducted using a 2-years landmark. Model was adjusted for age, sex, ethnicity, deprivation index, body mass index categories, multimorbidity (prevalent diabetes, hypertension, CVD and cancer) as well as long-standing illness , walking pace, sleep duration, watching TV, smoking and dietary intake (alcohol, fruits and vegetables, red meat, processed meat and oily fish intake), excluding the appropriated grouping variable .

Appendix Table S3. Hazard ratio (95% CI) for all-cause dementia mortality per 5kg lower handgrip strength stratified by subgroups

	N	Events	HR	Lower (95% CI)	Upper (95% CI)	P	P for interaction
Body mass index							
Normal-weight	153823	444	1.14	1.06	1.22	<0.001	0.626
Overweight	198786	558	1.20	1.13	1.27	<0.001	
Obesity	111775	298	1.12	1.04	1.21	0.004	
Walking pace							
Slow pace	36115	232	1.15	1.06	1.25	<0.001	0.846
Average pace	245255	700	1.12	1.06	1.18	<0.001	
Brisk pace	185418	377	1.25	1.16	1.35	<0.001	
Smoking status							
Never	256879	575	1.17	1.10	1.24	<0.001	0.035
Former	162225	611	1.15	1.09	1.21	<0.001	
Current	47684	123	1.26	1.12	1.43	<0.001	
Sleep time							
Normal sleep (7-9 h/d)	345140	958	1.16	1.11	1.22	<0.001	0.886
Short sleep (<7 h/d)	113707	284	1.19	1.09	1.29	<0.001	
Long sleep (>9 h/d)	7941	67	1.15	0.98	1.35	0.083	

Data presented as hazard ratio (HR, 95%CI). All analyses were conducted using a 2-years landmark. Model was adjusted for age, sex, ethnicity, deprivation index, body mass index categories, multimorbidity (prevalent diabetes, hypertension, CVD and cancer) as well as long-standing illness, walking pace, sleep duration, watching TV, smoking and dietary intake (alcohol, fruits and vegetables, red meat, processed meat and oily fish intake), excluding the appropriated grouping variable.
